# Expectations do not Influence the Response to Phosphosdiesterase Type 5 Inhibitor Therapy for Erectile Dysfunction

**DOI:** 10.3390/pharmacy3040295

**Published:** 2015-11-16

**Authors:** Connie C. J. Louizos, Peter K. Knight

**Affiliations:** School of Medical Sciences, University of Sydney, Sydney NSW 2006, Australia; E-Mail: conniecj@tpg.com.au

**Keywords:** patient expectations, erectile dysfunction, phosphodiesterase Type 5 inhibitors, questionnaire

## Abstract

It has been reported that patients frequently discontinue treatment for erectile dysfunction (ED) with phosphodiesterase type 5 inhibitors (PDE5Is) despite apparently good therapeutic results. Because expectations have been shown to affect patients’ appraisals of many drugs, the purpose of this study was to determine whether expectations affected the therapeutic response to PDE5Is in men with apparent psychogenic ED. An unvalidated questionnaire was used to collect data on expectations in 80 men commencing PDE5I therapy, and after three and six months of treatment. At the same time, subjects completed the International Index of Erectile Function (IIEF), the Sexual Excitation/Inhibition Scale (SIS/SES) and Beck’s Depression Inventory (BDI). No evidence of an effect on expectations on changes in IIEF or BDI scores could be identified. Although changes in IIEF, and BDI scores from recruitment to three months were indicative of improved sexual function and less depression, scores for most items on the expectations scale decreased, suggesting that expectations were not being met. The items for which scores decreased were the expectation to be prescribed a drug, that the drug would restore the sexual function to normal, would work within 30 minutes of administration, improve patients confidence to engage in sexual activity, and that the medication was the best treatment for ED across the three data collection points. The findings of this study indicate that improvements in erectile function did not translate into changes in medication expectations that suggested user satisfaction.

## 1. Introduction

Phosphodiesterase Type 5 Inhibitors (PDE5Is) are an established first line treatment for erectile dysfunction (ED). However, discontinuation rates ranging from 15% to 57% have been reported amongst PDE5I users, often despite good therapeutic outcomes [1,2,3,4,5]. The reasons for the high discontinuation rates have not been fully explained. 

Expectations and beliefs contribute to the therapeutic effect in patients being treated with a variety of drugs for many different conditions [6], and may therefore play a role in determining the response to PDE5Is. Expectations and beliefs, which reflect the patient’s perceptions of the aetiology, likely duration, and personal consequences of the condition being treated, can have positive or negative impacts on drug effects at several stages in the therapeutic process [7,8]. Evidence supporting the importance of expectations in shaping the response to medication comes from studies that have examined the placebo effect. A number of randomised controlled trials of PDE5Is have identified placebo responses in ED patients [9,10,11,12,13]. The positive placebo responses may have resulted from anticipation of future positive outcomes, reductions in anxiety or the activation of reward mechanisms [14]. However, high expectations may adversely impact a patient’s ongoing appraisal of a drug, affecting compliance and discontinuation rates. The decision to discontinue use can be explained by the disconfirmation theory, which states that consumers compare their pre-use expectations of products, including medications, with subsequent perceived performance [15,16]. A mismatch results in disconfirmation, which is an important trigger of dissatisfaction [17]. It has been shown that patients may respond to disconfirmation by changing medications or discontinuing their use [17,18,19,20]. Excessively high expectations may result in a perceived lack of efficacy which underpins the decision of the patient to discontinue use of the medication [21]. This may be particularly relevant to PDE5Is, as most patients have high, often unrealistic, expectations of the benefits of the medications [22]. 

Given that patient expectations of treatment have been shown to influence the therapeutic response to some drugs, the purpose of this study was to the explicitly explore whether the therapeutic response to PDE5Is could be predicted by an assessment of a patient’s expectations at the beginning of treatment. A second aim was to examine how expectations change over a period of treatment, as changing expectations may impact on a patient’s decision to continue to use the drug in the long term. 

## 2. Experimental Section

### 2.1. Materials and Methods

Approval for this study was obtained from the University of Sydney Human Research Ethics Committee (approval 2012/458). The participants in this study, who gave informed written consent to participate, were patients of 13 medical general practitioners (GPs) practicing in Australia. The GPs had identified themselves as having a special interest in the treatment of erectile problems by enrolling on the online register of practitioners maintained by Impotence Australia [23]. There are no requirements for registration, and the register is available to the public. 

All men who consulted a participating GP and met the inclusion criteria were invited to enrol in the study. In order to be included, men had to be diagnosed with psychogenic ED and to have never previously been treated with PDE5Is. The clinical diagnosis was based on the absence of evidence of any vasculogenic or other physical cause of ED, and was reached using the protocols normally followed by the examining GPs. The diagnosis was not based on the results of tests conducted as part of a specialist investigation, nor did it specifically comply with the requirements for a diagnosis of psychogenic ED specified in the Diagnostic and Statistical Manual of Mental Disorders. Men who at the time of recruitment were identified as having any comorbidity that could contribute to ED, including use of specific medications, known drug or alcohol dependence, cardiovascular diseases or previously identified endocrinological abnormalities were excluded from the study. Participants were required to be between the ages of 18 and 70 years, in a stable heterosexual relationship of at least six months duration, and be fluent in English. There was no requirement for participants to be making a minimum number of attempts to achieve intercourse over the course of the study.

Recruitment continued until the target of 100 enrolled subjects was reached. Each GP was asked to recruit as many men as possible. At recruitment, each participant completed the expectations questionnaire described below. They also completed the International Index of Erectile Function (IIEF) [24], the Sexual Inhibition/Sexual Excitation Scale (SIS/SES) [25], Becks Depression Inventory (BDI) [26], and a general questionnaire eliciting basic demographic information. 

The IIEF is a 15 item self-report questionnaire that has been used extensively in the assessment of erectile dysfunction. IIEF items are categorised into five domains: erectile function (IIEF-EF), orgasmic function (IIEF-OF), sexual desire (IIEF-SD), intercourse satisfaction (IIEF-IS), and overall satisfaction (IIEF-OS). The SIS/SES is a 45 item self-administered questionnaire that examines responses to stimuli that could elicit sexual inhibition and sexual excitation. The questions describe hypothetical situations, some of which incorporate elements of threat. The SIS/SES yields three total scores: Sexual Excitation (SES), Sexual Inhibition 1 (SIS1—threat of performance failure), and Sexual Inhibition 2 (SIS2—threat of consequences). The BDI is a self-report 21-item scale that assesses the severity of symptoms associated with depression to enable a numerical depression score to be calculated.

At the completion of the first visit, participants were issued a prescription for a PDE5I and appointments were made for repeat consultations to occur three and six months after the date of recruitment. At these consultations the questionnaires were again completed. 

### 2.2. Expectations Questionnaire

A search of the Medline database using combinations of the keywords erectile dysfunction, satisfaction, expectations, phosphodiesterase type 5 inhibitors and questionnaires failed to identify any questionnaires suitable for investigating expectations for PDE5I therapy. Consequently, it was necessary to develop a new instrument. 

The process began with the generation of an initial list of questions by the authors that was based on the themes identified in the literature search. Questions examining general attitudes towards medications, and the subjects’ perceived understanding of how PDE5Is should be used were included as such knowledge has been shown to influence satisfaction with other drugs [27]. The initial list contained 19 questions, seven of which were removed following an initial review which assessed relevance and overlap in content between questions which was conducted by the authors. The 12 remaining questions (expectation items) were formatted as 5-point Likert scales, with responses assigned a numerical value for analysis. The response options (with the value assigned to them) were: strongly disagree (1), disagree (2), neutral/unsure (3), agree (4), and strongly agree (5).

The questionnaire was tested by administering it to 10 ED patients visiting a GP, who were asked to provide written feedback on its ease of use and suggestions for how the clarity of questions or instructions could be improved. The only suggestion received was to simplify the instructions for completing the questionnaire. The final items in the questionnaire are shown in Table 1.

**Table 1:** Items on the expectations questionnaire with mean (± sd) scores at recruitment and after three and six months of PDE5I treatment.

		Recruitment	3 months	6 months
1	I expected to be prescribed a drug when I decided to visit the doctor with this problem.	3.49 ± 1.07	3.14 ± 1.06 ^b^	1.49 ± 0.55 ^b,d^
2	Erectile dysfunction is having a significant adverse effect on my life.	1.73 ± 0.57	1.83 ± 0.85	1.48 ± 0.53 ^a,c^
3	I expect that this medication will restore my sexual function to normal.	3.13 ± 0.79	2.76 ± 1.12^a^	1.34 ± 0.58 ^b,d^
4	I expect that this medication will work within 30 minutes of taking it.	2.06 ± 1.11	1.93 ± 1.05	1.36 ± 0.51 ^b,d^
5	I expect that this medication is the best treatment available for erectile dysfunction.	3.10 ± 0.84	2.83 ± 1.06 ^a^	1.78 ± 0.91 ^b,d^
6	I expect that the effects of this medication will last long enough for me to complete intercourse.	3.09 ± 0.77	2.74 ± 0.92 ^a^	2.99 ± 0.95
7	I expect that this medication will increase my confidence to engage in sexual activity	2.02 ± 0.66	1.82 ± 0.48 ^a^	1.44 ± 0.91 ^a,c^
8	I expect that my partner will be satisfied with the effects of my treatment.	1.93 ± 1.09	1.74 ± 1.02 ^b^	2.71 ± 1.30 ^b,d^
9	In the past, I have had generally good experiences and results with medications.	2.05 ± 0.59	1.67 ± 0.53 ^a^	1.69 ± 0.57
10	I believe that I have sufficient understanding of how the medication is used.	1.31 ± 0.67	1.20 ± 0.43	1.51 ± 0.60 ^a,c^
11	I believe that I have sufficient understanding of the medication’s effects.	1.85 ± 0.51	1.61 ± 0.51 ^a^	1.84 ± 0.93
12	I believe it is better to do without medications.	1.40 ± 0.80	1.28 ± 0.55	1.27 ± 0.55

Notes: ^a^ Significantly different from recruitment score *P* < 0.05; ^b^ Significantly different from recruitment score *P* < 0.01; ^c^ Significantly different from 3-month score *P* < 0.05; ^d^ Significantly different from 3-month score *P* < 0.01.

The initial decision to recruit 100 participants was arbitrary. The decision was tested using a post hoc power analysis (G Power 3.1) assessing the ability to detect a 6-point change in IIEF-EF score, which is considered the smallest change to be clinically relevant [24].

Due to difficulty in recruiting participants for the study, it was not possible to validate the questionnaire before administration, as validation requires a unique group of subjects whose results cannot be used in the experimental study.

### 2.3. Statistical Analysis

All statistical analyses were conducted using IBM SPSS version 20. Descriptive statistics (mean and standard deviation) were calculated for responses obtained from the expectations, IIEF, SIS/SES, BDI and demographic questionnaires. Skewness and kurtosis were also calculated as indicators of the normality of the data distribution. Data that were not normally distributed were log transformed before analysis. All means and standard deviations reported below are for untransformed results. 

Recruitment IIEF, SIS/SES and BDI scores for subjects prescribed sildenafil and tadalfil were compared using unpaired t tests. As no difference was found between groups, the results were pooled for analysis. In order to minimise heterogeneity of the subjects, only those receiving the highest possible dose were included in the analyses reported below.

Pearson product-moment correlation coefficients were calculated for items on the expectations scale. Correlations between the following data sets were calculated: expectation, IIEF and BDI scores at recruitment; expectations scores at recruitment and IIEF and BDI changes 0–3 months; expectations scores at recruitment and IIEF and BDI changes 0–6 months; changes in expectations scores and changes in IIEF and BDI scores 0–3 months; and changes in expectations scores and changes in IIEF and BDI scores 3–6 months. Changes in SIS/SES scores were not analysed in this way, as the scale may measure underlying traits rather than time specific patient states [25].

Repeated measures ANOVA was used to identify significant changes in scores for the expectations, IIEF, SIS/SES and BDI scales between the three data collection points. 

For all analyses described above, statistical significance was defined by α < 0.05.

## 3. Results

### 3.1. Descriptive Statistics

Details of the specific PDE5I prescribed and the dose rate at which it was to be used were not obtained for 17 men. All but three of the remaining 83 subjects were initially prescribed, and continued to use, the highest dose of PDE5I, with 59 men receiving 100 mg sildenafil and 21 men 20 mg tadalafil. Results for the 17 recruited subjects for whom drug information was not available, and the three that did not receive the highest dose were not included in the analyses.

The mean age at recruitment of the 80 subjects included in the study was 52.2 ± 6.2 years (range 40–67 years). Mean scores on the IIEF, SIS/SES and BDI at recruitment, and the IIEF and BDI at the 3- and 6-month data collections are shown in Table 2. There was a significant increase in scores in each of the IIEF domains from recruitment to the 3-month data collection, and a further increase from 3–6 months. Significant decreases in BDI scores were observed from recruitment to three months, and from 3–6 months.

**Table 2:** Mean (±sd) scores on the IIEF, SIS/SES and BDI scales at recruitment and after three and six months of PDE5I treatment.

	Recruitment	3 months	6 months
IIEF-EF	8.77 ± 2.16	16.33 ± 4.65 ^a^	21.41 ± 2.73 ^a,c^
IIEF-OF	3.73 ± 1.21	4.98 ± 1.73 ^a^	6.29 ± 0.88 ^a,c^
IIEF-SD	3.41 ± 1.43	5.55 ± 1.77 ^a^	7.59 ± 1.01 ^a,c^
IIEF-IS	2.81 ± 0.91	5.23 ± 1.74 ^a^	9.38 ± 1.61 ^a,c^
IIEF-OS	2.56 ± 0.57	5.23 ± 1.59 ^a^	6.22 ± 0.94 ^a,c^
BDI	23.26 ± 7.35	11.68 ± 4.72 ^a^	10.19 ± 3.18 ^a,b^
SES	53.87 ± 4.11		
SIS1	38.77 ± 4.06		
SIS2	35.35 ± 4.00		

^a^ Significantly different from recruitment score *p* < 0.01; ^b^ Significantly different from 3-month score *p* < 0.05; ^c^ Significantly different from 3-month score *p* < 0.01

At recruitment, 75% of participants had BDI scores in the range 19–29, indicating moderately severe depression, with a further 12% scoring 30 or greater—indicative of severe depression [28]. At three months, 6% of participants had scores in the moderate range, with none in the severe range. At six months, 40% of participants scored in the moderate range, and 5% in the severe range. The percentage of participants whose scores indicated that they were not depressed increased from 9% at recruitment to 33% at three months and 44% at six months.

Mean scores for the expectations items at the three data collection points are shown in Table One. Significant decreases in scores from recruitment to three months were observed for all questions except 2, 10 and 12. At six months, scores for questions 1, 2, 3, 4 and 5 were significantly lower than the 3-month scores, whereas the scores for questions 8 and 10 were significantly higher. The score for question 10 was also higher than at recruitment.

### 3.2. Correlations

Because of the large number of correlation analyses conducted, only significant results are presented here.

The following correlations were observed between expectations item scores and questionnaire scores at recruitment: Item 2 and SIS2 (0.30, *p* < 0.01); Item 8 and IIEF-EF (0.27, *p* < 0.05); Item 8 and BDI (−0.28, *p* < 0.05); Item 10 and SES (0.28, *p* < 0.05); and Item 12 and BDI (−0.40, *p* < 0.01).

The change in IIEF-SD score from 0–3 months was correlated with recruitment scores for Item 1 (r = −0.24, *p* < 0.01); Item 3 (r = 0.23, *p* < 0.01); Item 8 (r = 0.35, *p* < 0.01); Item 10 (r = 0.23, *p* < 0.01) and Item 12 (r = 0.25, *p* < 0.01). The change in IIEF-IS score from recruitment three months correlated with the recruitment score for Item 4 (r = 0.24, *p* < 0.05) as was the change in IIEF-OS score (r = 0.25, *p* < 0.05). The change in BDI score from recruitment to three months was correlated with the recruitment scores for items Item 5 (r = 0.40, *p* < 0.01); Item 6 (r = 0.33, *p* < 0.01) and Item 12 (r = 0.30, *p* < 0.01).

The change in IIEF-SD score over the six months of the study correlated with the recruitment scores for Items 1 (r = −0.34, *p* < 0.01) and 8 (r = 0.27, *p* < 0.05). The change in IIEF-OS was correlated with recruitment scores for Items 4 (r = 0.40, *p* < 0.01) and 11 (r = −0.26, *p* < 0.05); and the change in BDI with Items 5 (r = 0.28, *p* < 0.05) and 12 (r = 0.34, *p* < 0.01).

The only significant correlation between changes in expectation item and questionnaire scores from 0–3 months was IIEF-SD and Item 2 (r = −0.28, *p* < 0.05). The following significant correlations were identified between changes in scores for expectations items and changes in questionnaire between three and six months: IIEF-EF and Item 10 (r = 0.29, *p* < 0.05); IIEF-OF and Item 6 (r = 0.35, *p* < 0.01); IIEF-SD and Item 4 (r = 0.35, P < 0.01); IIEF-IS and Items 4 (r = 0.33, *p* < 0.01) and 11 (r = 0.25, *p* < 0.05). 

We originally intended to perform multiple linear regression is in order to identify predictors of the changes occurring in response to treatment. However the low number of significant correlations between variables rendered the data unsuitable for this analysis.

### 3.3. Power Analysis

The post hoc power analysis yielded a value of 73%, with a minimum detectable difference of 5.75 and a required sample size of 58.

## 4. Discussion

This paper reports findings relevant to the prescription of PDE5Is in general medical practice and reflects routine clinical practice at the GP level of care. It is important to emphasise that the diagnosis of ED was based on IIEF cutoffs, and that a definitive diagnosis of psychogenic ED was not made. Rather, the diagnosis was presumptive and based on the absence of causes of ED that were identifiable on routine clinical examination. The diagnosis therefore depended on the examination conducted by each of the doctors, which could not be controlled. Although there are established guidelines for the investigation of ED, there is little evidence confirming that these are adhered to in general practice. One study found that GPs treating men presenting with ED took a sexological history in only 85% of cases, and requested laboratory tests in 13.6% [29]. The authors suggested that this lack of thorough investigation may reflect the fact that “men may in many cases request a drug prescription from their GP and may not be interested in a physical examination”. A separate study found that 83.9% of GPs believed that only selected patients required extensive diagnostic evaluations for ED [30]. Even amongst urologists there is evidence that investigations of conditions potentially contributing to ED are often abbreviated or deferred [31]. 

The first aim of this study was to determine whether expectations influence the response to PDE5Is. The paucity of correlations between expectations and measures of sexual function meant we were unable to fully explore this question, nor to address the question of what influences expectations. On the basis of the results of this study there is no evidence to support the view that expectations influence the therapeutic response to PDE5Is.

Across the period of the study, the increases in IIEF scores indicated improved erectile function and sexual satisfaction. The mean increase in IIEF scores from recruitment to three months exceeded the six point increase that is considered necessary for clinical significance [24]. While it is important to recognize that patient satisfaction with the medication was not directly measured, overall agreement with items 5, 6, 7 and 8 on the expectations questionnaire decreased from recruitment to three months (during which time overall sexual satisfaction measured on the IIEF-OS scale increased significantly). Given the nature of the questions, and interpreting these findings in terms of disconfirmation theory suggests that the changes in erectile function were insufficient to satisfy the patients. One possible explanation of the observed findings is that patient expectations for the effects of medication were excessively high. This is reflected in the response to the statement “I expect that this medication will restore my sexual function to normal” which had the second highest level of agreement of all statements at recruitment. It is likely that this is an unrealistic expectation, especially amongst men with apparent psychogenic ED, a condition for which combined psychological and pharmaceutical therapy gives better outcomes than medication use alone [32]. 

Hypothesising that expectations were excessively high raises the question of “how high is too high?”. Only four items at recruitment had expectation scores greater than 3 (indicating overall agreement). It therefore appears that low expectation scores do not preclude the possibility that expectations will be too high to be met. An alternative approach is to ask “how much improvement in erectile function is required before patients are satisfied”—a question that cannot be answered from this study but which may be important in improving satisfaction with PDE5I use. 

Expectations had not stabilised after three months of treatment. Between three and six months, the expectation to be prescribed a medication, the expectation that sexual function would be restored to normal and the expectation that the medication was the best treatment for ED all decreased. This suggests an ongoing loss of confidence in the drug despite improving erectile function, and may provide some explanation for the reported high discontinuation rates for PDE5Is [33]. 

In the 3–6 month period there were significant increases in scores for items 8 and 10 which relate to the patient’s perception of partner satisfaction and understanding of drug use. Agreement with both statements was higher at six months than at either of the earlier data collection times. At three months, agreement with the statement that “ED is having a significant adverse effect on my life” was higher than at recruitment, but by six months agreement was significantly lower than the recruitment score. This change mirrors the change in agreement with the statement on perceived partner satisfaction. Taken together, the findings show that although the initial response to ED medication may be disappointing, there is scope for improvement in some domains if treatment is continued for long enough. However, with the exception of IIEF-EF, the size of the changes in domain scores required for a clinically significant outcome are unknown, and it is therefore impossible to determine whether the observed changes are of significance to the patient. The low scores for item 10 indicate that patient education was less than optimal, and that to some extent, men appear to learn how to use the medications by experience. 

A review of the correlations between items sheds some light on the relationships between expectations and sexual confidence in this group of men. There was strong negative correlation between the expectation that the drug would restore sexual function to normal and that it would increase the users’ confidence to engage in sexual activity. This suggests that improved physical capacity for intercourse was less important than other factors in generating their sexual confidence, and supports the view that in cases of psychogenic ED, the use of medications to restore erectile function should not be the only therapeutic strategy employed [34]. The importance of a rapid onset of action is reflected in its moderate positive correlation with the expectation that the drug will increase sexual confidence—in other words, a faster acting drug will have a greater beneficial effect on confidence. However, a rapid onset of action is more strongly correlated with expectations that partner satisfaction will increase. 

A large number of participants met the BDI criteria for depression. Despite receiving no specific pharmacological or psychological treatment for depression, mean BDI scores decreased from recruitment to three months, with a further decrease from three to six months. The finding that men in this study were depressed is in accordance with other studies that have reported a relationship between depression and ED [35,36]. However, the nature of the relationship between the two conditions is unclear as ED may contribute to, or be a consequence of, depression [37]. The fact that the changes in IIEF-EF and BDI scores were not correlated suggests that depression was not a significant contributor to the aetiology of ED in these men. This contrasts with the previously reported negative correlation between changes in IIEF-EF and depression scores in men with ED who received treatment with PDE5Is [36]. However, men with ED of any aetiology were included in that study, and it is possible that the relationship between ED and depression varies according to the underlying cause of the ED. A positive effect of PDE5I therapy on depression has also been previously reported. Significantly greater reductions in symptoms of depression were reported in ED patients being treated with vardenafil compared with subjects in placebo groups [36,38] and changes in IIEF scores were negatively correlated with changes in scores on a depression scale in men with ED who received treatment with PDE5Is [36]. However, there is evidence of a placebo effect on changes in BDI. At the end of a six week double-blind study 73.2% of subjects treated with sildenafil, who were clinically depressed at recruitment, had a BDI score indicative of no or minimal depression. However, 47.1% of the placebo were not depressed at the end of the study [39]. Orr *et al.* [40] observed a positive effect of sildenafil on depressive symptoms independent of changes in EF. They suggested that these could be due to the effects of the drug on the autonomic nervous system or hippocampal neurogenesis. Whilst the findings of the present study have again shown a relationship between treatment with PDE5Is and a decrease in depressive symptom, they do not shed further light on the mechanism by which this effect is mediated. Changes in IIEF variables were not correlated with the observed improvement in BDI score, supporting the view that changes in EF are not principally responsible for improvements in depressive symptoms. 

The study has several limitations that must be considered. The first is the fact psychogenic ED was a diagnosis of exclusion, with none of the subjects undergoing the full battery of tests necessary to achieve a definitive diagnosis. The results of the study do however reflect routine non-specialist medical management of ED patients. The study was observational with no control group, and patient history and clinical intervention could not be controlled. The possibility of a participation bias cannot be excluded. Because recruitment was limited to subjects diagnosed with apparent psychogenic ED, we do not suggest that the findings would be translatable to men with other types of ED.

A second important limitation is the fact that the expectations questionnaire was not validated. The decision not to validate the questionnaire was a practical one, based on the difficulty of recruiting sufficient subjects to complete both validation and experimental phases.

## 5. Conclusions

In conclusion, although expectations did not predict the response to therapy, the significant changes in expectations that were identified suggest the need for better patient management and counselling. Specifically, although erectile function improved with PDE5I use, agreement with statement that men understood how to use the drug remained low throughout the study. The study has shown the importance of advising men that initial dissatisfaction with the effects of PDE5Is on anticipated partner satisfaction and life impacts can be reversed if they persevere with treatment. There is a need to re-establish sexual norms after a period of ED, and initial attempts at intercourse are frequently associated with excessive anxiety [23]. As IIEF, BDI and several expectations scores changed from three to six months, it appears that men should persevere with PDE5I use for at least six months. A second important finding is that even after six months of use, agreement with the idea that the patient has sufficient knowledge of how PDE5Is are used remains low. Further examination of educational needs and how these can be met appears warranted. 
